# Reducing Loneliness Among Aging Adults: The Roles of Personal Voice Assistants and Anthropomorphic Interactions

**DOI:** 10.3389/fpubh.2021.750736

**Published:** 2021-12-10

**Authors:** Valerie K. Jones, Michael Hanus, Changmin Yan, Marcia Y. Shade, Julie Blaskewicz Boron, Rafael Maschieri Bicudo

**Affiliations:** ^1^University of Nebraska-Lincoln, College of Journalism and Mass Communications, Lincoln, NE, United States; ^2^University of Nebraska Medical Center, College of Nursing, Omaha, NE, United States; ^3^Department of Gerontology, University of Nebraska-Omaha, College of Public Affairs and Community Service, Omaha, NE, United States

**Keywords:** loneliness, aging, gerontology, personal voice assistant, anthropomorphism, artificial intelligence, Amazon Alexa, conversational agent

## Abstract

The perception of feeling lonely is an influential factor in determining quality of life among aging adults. As the US Census Bureau projects that the number of Americans ages 65 and older will double by 2060, reducing loneliness is imperative. Personal voice assistants (PVAs) such as Amazon's Echo offer the ease-of-use of voice control with a friendly, helpful artificial intelligence. This study aimed to understand the influence of a PVA on loneliness reduction among adults of advanced ages, i.e., 75+, and explore anthropomorphism as a potential underlying mechanism. Participants (*N* = 16) ages 75 or older used an Amazon Echo PVA for 8 weeks in an independent living facility in the Midwest. Surveys were used to collect information about perceived loneliness, and PVA interaction data was recorded and analyzed. Participants consistently exceeded the required daily interactions. As hypothesized, after the first 4 weeks of the intervention, aging adults reported significantly lower loneliness (baseline mean = 2.22, SD = 0.42; week 4 mean = 1.99, SD = 0.45, *Z* = −2.45, and *p* = 0.01). Four dominant anthropomorphic themes emerged after thematic analysis of the entire 8 weeks' PVA interaction data (Cohen's Kappa = 0.92): (1) greetings (user-initiated, friendly phrases); (2) comments/questions (user-initiated, second-person pronoun), (3) polite interactions (user-initiated, direct-name friendly requests), (4) reaction (user response to Alexa). Relational greetings predicted loneliness reductions in the first 4 weeks and baseline loneliness predicted relational greetings with the PVA during the entire 8 weeks, suggesting that anthropomorphization of PVAs may play a role in mitigating loneliness in aging adults.

## Introduction

One of the most influential factors in determining quality of life among aging adults is the perception of feeling lonely (1, 2). Loneliness refers to perceived isolation or the sense of lacking companionship, and the negative feelings that can arise from not having a companion or emotional support, or a perceived lack of wider social networks (3–5). The experience of loneliness has been associated with reduced opportunities for companionship, with older adults experiencing consequential social and emotional loneliness (6). Higher rates of depression, self-harm, self-neglecting behavior and mortality, as well as predictions of functional decline and death, have been associated with perceived loneliness (4, 7–9). Loneliness reduction is a pathway to improve aging adults' perceived life quality (2). When mitigated through technology such as Internet use, older adults reported improved quality of life (10). As the number of Americans ages 65 and older is projected to nearly double by 2060, to 95 million, the ability to reduce feelings of loneliness among aging adults in a cost effective, efficient manner is increasingly important (11).

Interventions to decrease loneliness in older adults have included companionship by ways of social facilitation, psychological therapies, health and social care provisions, animal interactions, and befriending, and increasingly, the introduction of information and communication technologies (ICT) (8, 12). These ICT interventions primarily involve training participants on an ICT device (most frequently, an Internet-enabled computer) and encouraging them to use the device to meet others, stay in contact with family, or engage in hobbies (10, 13, 14). Communication programs such as smartphones, iPads, email, and online chat rooms or forums, as well as technological innovations such as the Wii and virtual pet companions, have been found to have a positive influence on reducing loneliness (13). ICT interventions can reduce the cost of dedicated personnel visits and increase the opportunities one has for social connection; once the training period has ended, aging adults are free to use the device whenever and for as long as they desire. However, ICT devices, mainly computers and tablets, are restrictive to those who have difficulty typing, poor eyesight, or difficulty learning an unfamiliar system, which are common challenges in the aging adult population (15).

The introduction of personal voice assistant (PVA) devices or “smart speakers” such as the Google Nest or Amazon Echo provides a new opportunity for ICT intervention that may address drawbacks found in computer or tablet use. PVAs are essentially “voice assistants embodied in smart speakers” and have been labeled as “intelligent personal assistants, conversational agents, and virtual personal assistants” (16). These devices are highly accessible; they remove the physical requirements of viewing a screen and using a keyboard or touchscreen and are controlled by voice commands, which have been found appealing among older adults (15). Voice is quickly growing to become the predominant means of device interaction; 50% of searches were estimated to be done *via* voice in 2020 (17).

PVAs feature an interactive artificial intelligence (AI) that acts as an assistant who can respond, chat, or help at any time. While an AI cannot provide the same levels of conversation or support as a human visitor, research indicates that individuals view devices as possessing human-like qualities and can develop meaningful relationships with AI or other conversational bots (18, 19). This attribution of human traits to non-human entities is referred to as anthropomorphism (20). The phenomenon of anthropomorphizing AI technology is well-documented in both popular culture and research (21). There is also a strong body of evidence that humans anthropomorphize technology, including computers (22, 23), smartphones (24), cars (25), and robots (19, 26). Lonely individuals (i.e., those lacking social connection) are more likely to anthropomorphize non-human agents (27). One of the main motivations to anthropomorphize non-human entities is the desire to form social connections with non-human entities in the absence of humans (20, 28). Prior research demonstrated individuals who were more chronically disconnected from other humans were more likely to see their pets and other animals as having more traits related to social connection (e.g., thoughtful, considerate, and sympathetic) (28).

With the advent of PVAs, there is little surprise that people anthropomorphize PVAs as well (16, 29). Anthropomorphism for PVAs may be particularly strong, as PVAs are created to be social agents (e.g., a human voice from a PVA has been shown to increase social perceptions) (30). Adaptability, usefulness, enjoyment, sociability, perceived behavioral control and companionship are the variables that most indicate human acceptance of social robots, and PVAs are designed to exhibit all of these characteristics (31). With aging adults, as the number of relationships diminish, emotional connection through companionship strengthens with those remaining in a more limited social circle (32). In combination with relative affordability and accessibility, the AI-driven human voice and broad array of knowledge and programs make the PVA a prime candidate to create social connection—and in turn, elicit anthropomorphism—with the user. As a result, individuals who are lonely may turn to a PVA in order to gain social connection and feel less lonely.

Building on prior empirical studies of PVAs and aging adults which primarily focus on exploratory user experience such as how aging adults use PVAs (16, 33, 34) and how the PVAs provide companionship (35, 36), the current study aimed to investigate the impact of such PVA interactions on anthropomorphization and loneliness reduction. Despite commercial interests in PVAs and loneliness mitigation in aging adults from organizations such as the American Association of Retired Persons (37) and The Abbeyfield Society in the U.K. (38), there is a knowledge gap about PVA's efficacy on loneliness reduction and the pathway to such potential effects.

With voice commands becoming increasingly common and responsive, and intuitive AI becoming increasingly smarter, a PVA in the home could be a means of breaking through barriers of other ICT interventions and providing substantial benefits to an older population. Further, living alone could make aging adults, especially the understudied “older old” of adults 75+, particularly motivated to forge social bonds with AI technology. The Pew Research Center Social and Demographic Trends 2009 survey results suggest 75 is a significant turning point for older Americans (65 or older) to experience feeling old and other life changes such as “failing health, an inability to live independently, an inability to drive, difficulty with stairs” (39). However, there is limited existing research that investigates loneliness outcomes of PVA use among this population. The purpose of this study, then, was to explore the influence of a PVA on loneliness reduction among aging adults 75+ living alone, and the role of anthropomorphic interaction with AI. Therefore, we hypothesized the following outcomes regarding loneliness reductions, anthropomorphization as a potential mechanism, and loneliness-driven anthropomorphic interactions:

H1: There will be significant reductions in loneliness among aging adults living alone in the first 4 weeks of the Alexa PVA intervention.

H2: Anthropomorphic interactions with the Alexa PVA will predict reductions in loneliness among aging adults living alone in the first 4 weeks of the Alexa PVA intervention.

H3: Baseline loneliness will predict anthropomorphic interactions with the Alexa PVA among aging adults living alone during the entire 8-week intervention.

## Methods

### Study Design, Sample, and Procedures

This was a single-group quasi-experimental study design approved by the IRB. Adults 75 years of age and older were recruited from an independent living facility in the Midwest through flyers and informational presentations. In order to qualify for the study, participants had to live in their apartments alone (i.e., not with a spouse, relative or someone else), be fluent in English, have normative cognitive functioning (evaluated *via* an abbreviated mini-cognitive assessment over the phone (40), and could not currently own an Amazon Echo Dot or Google Home.

Researchers set up the Echo in participants' homes and trained them on how to use it. During the study period, participants were required to interact with the device at least five times each day, choosing commands from a provided list of 100 commands. The list was developed based on prior research about common uses of Alexa (41, 42). Selecting the commands allowed participants some agency and control, appreciated by older adults (43). Researchers monitored device usage, and gave reminders if participants did not meet requirements on days 7, 14, and 21. Starting at week 5, participants were allowed to use the device as much or as little as they wished. Such a study design ensures “minimal intervention needed to produce change” (44) will be met during the first 4 weeks *via* mandatory minimum interactions with the device while allowing participants to interact with the device voluntarily in a naturalistic way during the second 4 weeks.

To assess how participants used the PVA, it was important to have a time-stamped record of every interaction with the device and later be able to categorize the types of requests from the participants. These PVAs are designed by Amazon to record requests after hearing the wake word “Alexa,” and send the requests to Amazon's secure cloud, where they are accessible through connected accounts (45). With participants' permission, each PVA device was linked with two accounts: the researchers' and the participant's. Only the participant had access to their participant account (i.e., researchers assisted them in creating the account, but participants created a password that the researchers did not know). Both accounts enabled access to device-usage data, which included every interaction the participant had with the Echo. As participants used their Echo, their usage data was recorded and linked to their account (e.g., if someone says “Alexa, what's the weather today” the device logged the time and what was asked). Following the conclusion of the study, the researchers copied all the interaction data from the device over the study period and then deleted the researchers account from the device, preventing them from seeing any future interaction data. Participants could continue to use their device uninterrupted through their participant account.

A manipulation check was performed to track the number of daily interactions to ensure participants had sufficient interactions with the device. In the first 4 weeks during which a minimum of five interactions were required, participants reported an average of 18 daily interactions with the device. During the second 4 weeks, they reported an average of 10 daily interactions with the device, even when no minimum interactions were required. Therefore, participants had sufficient interactions with the intervention device.

### Measurement

Measurement consisted of survey items assessing perceptions of loneliness immediately before the study (baseline), after 4 weeks (week 4) of use, and a data log that recorded all participant interaction with the PVA during the entire 4 weeks. Participants' computer usage and usage of any apps on a smartphone in the week prior to the study were measured on a 4-point scale (1: <1 day, 2: 1–2 days, 3: 3–4 days, 4: 5–7 days).

#### Loneliness

Loneliness was measured by an abridged eight-item UCLA loneliness scale designed for remote assessment (16, 46, 47) immediately before the intervention and after 4 weeks during which participants were required to complete at least five daily tasks on the PVA. The items were assessed on a five-point scale ranging from one (strongly disagree) to five (strongly agree). Sample items include “I lack companionship,” “There is no one I can turn to,” and “I am no longer close to anyone” (reverse coded). Baseline and week 4 loneliness perceptions were each calculated by averaging the eight items. Cronbach's alpha was 0.77 for baseline loneliness and 0.67 for week 4 loneliness.

#### Anthropomorphic Interactions With the PVA

Anthropomorphic Interactions with the PVA for this study were determined using a thematic analysis, and operationalized as behaviors generally attributed to humans that demonstrated relational closeness, politeness, and interaction rituals. Anthropomorphic interactions were measured by extracting all recorded user commands among the 16 participating aging adults living alone and then coding for anthropomorphic themes during the first 4 weeks and the entire 8 weeks. The device-usage data recorded every interaction the participant had with the Echo. Only primary commands were included (incomprehensible commands, and deactivation and activation commands were excluded) (16).

### Data Analysis

Descriptive statistics and normality tests (48) were performed for the dependent variables (i.e., perceptions of loneliness at the baseline and after 4 weeks of use). To test H1, a two-tailed Wilcoxon signed-rank nonparametric test was performed at the 95% confidence interval to compare baseline and week 4 perceptions of loneliness.

To test H2 and H3, a thematic analysis (49) of the qualitative data from user interactions with the PVA was first performed to identify dominant themes and quantify individual anthropomorphic interactions under each theme. Using thematic analysis (49), anthropomorphic interactions were first extracted from all PVA interactions. Two coders looked for PVA interactions that exhibit behaviors generally attributed to humans that demonstrated relational closeness, politeness, and interaction rituals. A total of 901 anthropomorphic interactions were extracted. An inductive thematic analysis of anthropomorphic interactions (49) was then performed to identify patterned responses based on prevalence of repeated key words and significant meanings representing different types of relational closeness, politeness, and interaction rituals. Four dominant themes related to anthropomorphism emerged: relational greetings, comment/questions, polite behaviors and reactions. About 20% of anthropomorphic interactions were used to calculate intercoder reliability (Cohen's Kappa = 0.92). One coder coded the remaining data.

**Theme (1) Relational greetings** (user-initiated, friendly phrases).

This consisted of specific greetings to Alexa that one would typically use with a human companion. Participants' greetings included “Good morning,” “Hello, Alexa,” “Alexa, I'm going down for supper,” “Alexa, I'm home,” and “Good night.”

**Theme (2) Comments/questions** (user-initiated, second-person pronoun).

This included interactions in which the user was speaking directly to the device, asking about it or addressing it as an actual person or being. Participants' comments and questions typically included using “you,” e.g., asking “Alexa, how old are you?,” “Alexa, what can you do for me?,” “Alexa, do you have a poem you can quote for me that would relax me?,” “Alexa, what are you thankful for?,” “Alexa, I have been ignoring you, I'm sorry” and “You are in charge of the cat now, I'm leaving.”

**Theme (3) Polite behaviors** (user-initiated, direct-name friendly requests).

This included terms with user requests or commands that reflected politeness norms typically incorporated in conversations with people. Participants' polite interactions included “Alexa, can I hear some harp music?,” “Alexa, tell me a joke, please,” “Alexa, please play some lullabies,” or “Alexa, please let me know when it is four o' clock.”

**Theme (4) Reactions** (user response to Alexa).

This consisted of verbal responses to Alexa's responses or feedback. Participants' verbal reactions included “That's very good,” “I'm sorry, I can't think,” “Alexa, that's enough,” or “That was fun, thank you.”

Anthropomorphic interactions in the first 4 weeks, along with prior computer use and app use, were entered as predictors of reductions in loneliness in a multiple regression analysis to test H2. To test H3, a multivariate analysis of variance (MANOVA) was then performed to estimate a single regression model with baseline loneliness as the predictor, and numbers of anthropomorphic interactions within each theme throughout the entire 8 weeks as the response variables.

## Results

### Participant Characteristics

We conducted an 8-week within-subjects examination of *N* = 16 individuals, with ages ranging from 77 to 96 (M = 85.2 SD=5.02) living as the sole household resident. Of the participants, 69% were female and 31% male; 94% were white and 6% were black; and 12.5% had never married and 87.5% had been married.

#### H1 Loneliness Reductions

There will be significant reductions in loneliness among aging adults living alone in the first 4 weeks of the Alexa PVA intervention.

Table 1 includes a description of the baseline loneliness perceptions and the loneliness perception after 4 weeks of PVA use by the older participants.

**Table 1:** Descriptive statistics and normality tests of perceptions of loneliness.

**Variables**	**Mean**	**Median**	**SD**	**Skewness**	**Kurtosis**	**Shapiro–Wilk test**
Baseline loneliness	2.22	2.31	0.41	−1.65, SE = 0.56	3.92, SE = 1.09	*W*_(16)_ = 0.86, *p* = 0.018
Week 4 loneliness	1.99	2.13	0.45	−1.16, SE = 0.56	0.09, SE = 1.09	*W*_(16)_ = 0.81, *p* = 0.004

**Figure 1:**
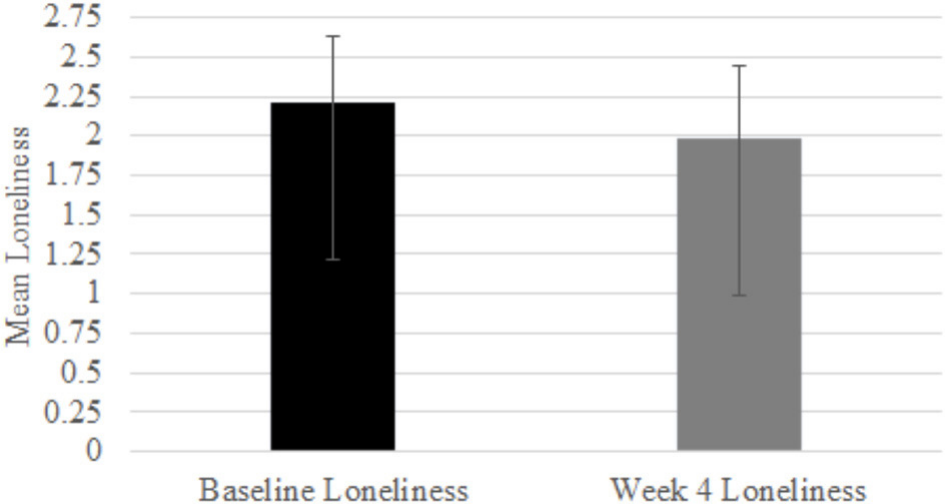
Self-reported loneliness comparison.

Based on the Shapiro–Wilk test results reported in Table 1, the null hypotheses of normal population distributions for perceptions of loneliness were rejected for both baseline [*W*_(16)_ = 0.86, *p* = 0.018] and week 4 [*W*_(16)_ = 0.81, *p* = 0.004] perceptions of loneliness at α = 0.05. The 2-tailed Wilcoxon signed-rank test showed that participants reported significant reductions in perceived loneliness after 4 weeks of using the PVA (*Z* = −2.45, *p* = 0.01; baseline mean = 2.22, SD = 0.42; week 4 mean = 1.99, SD = 0.45; see Figure 1), supporting H1.

#### H2: Anthropomorphization as a Potential Mechanism

Anthropomorphic interactions with the Alexa PVA will predict reductions in loneliness among aging adults living alone in the first 4 weeks of the Alexa PVA intervention.

Multiple regression was employed to examine the four themes of anthropomorphic interactions in the first 4 weeks as predictors of loneliness reductions during the same 4 weeks, controlling for prior week's computer use and app use in a single model. Overall the predictive model was significant, *F*_(6, 9)_ = 7.02, *p* < 0.005, Adjusted R Square = 0.71. Participants' 4-week loneliness reductions were significantly predicted by the number of greetings (β = 1.08, *p* < 0.05). However, the number of reactions (β = 0.10, *p* = 0.68), polite interactions (β = 0.03, *p* = 0.91), or comments/questions (β = −0.43, *p* = 0.25) did not significantly predict 4-week loneliness reductions. Neither prior week's computer use (β = −0.10, *p* = 0.61) nor app use (β = −0.39, *p* = 0.06) predicted 4-week loneliness reductions. Therefore, H2 was partially supported: relational greetings to the Alexa PVA predicted 4-week loneliness reductions.

#### H3: Baseline Loneliness as a Driver for PVA Anthropomorphic Interactions

Baseline loneliness will predict anthropomorphic interactions with the Alexa PVA among aging adults living alone during the entire 8-week intervention.

MANOVA was performed to estimate baseline loneliness as the predictor of each of the four themes of anthropomorphic interactions in the entire 8 weeks in a single model.

Overall the predictive model was significant, Wilks' Lambda = 0.40, *F*_(4, 11)_ = 4.09, *p* < 0.05, partial η^2^ = 0.60. Participants' baseline loneliness significantly predicted the number of greetings, *F*_(1, 15)_ = 10.08, *p* < 0.01, partial η^2^ = 0.42, adjusted *R* Squared = 0.38 (*b* = 43.34, *p* < 0.01). However, participants' baseline loneliness did not significantly predict the number of reactions, *F*_(1, 15)_ = 0.31, *p* = 0.59 (*b* = 0.93, *p* = 0.59), polite interactions, *F*_(1, 15)_ = 2.19, *p* = 0.16 (*b* = 10.08, *p* = 0.16), or comments/questions, *F*_(1, 15)_ = 1.70, *p* = 0.21 (*b* = 8.53, *p* = 0.21). Therefore, H2 was partially supported: baseline loneliness predicted relational greetings to the Alexa PVA.

## Discussion

This pilot study was designed to observe if PVAs can reduce loneliness for “older old” adults 75+ living alone and explore anthropomorphism as an underlying mechanism for loneliness reductions. The results provide preliminary evidence that a PVA can be regularly used by older individuals and may help reduce perceptions of loneliness within 4 weeks of use. We also found that baseline loneliness was the primary predictor to initiate friendly phrases to greet the PVA device during the 8 weeks of the intervention, suggesting that the lonelier an aging adult feels, the more likely she/he is going to treat PVAs as human, in anthropomorphic ways. Our findings are consistent with previous studies in which aging adults 65 or older personify PVAs by categorizing the devices as human-like and finding companionship through such interactions (16, 50). Results of the current study advance our understanding of PVA personification among aging adults by demonstrating the direct impact of such personification, i.e., as the aging adult anthropomorphizes PVAs, her/his loneliness subsides. In addition, our data illustrate a novel effect of baseline loneliness as an impetus for aging adults to anthropomorphize PVAs, perhaps as a mechanism to chip away isolation in her/his life.

As hypothesized, one of the main preliminary findings in this study was a decrease in the older adult participants' perceived loneliness after use of the PVA. This supports prior research suggesting that new technologies can provide promising opportunities for addressing loneliness in aging adults (51), and demonstrates how ICT interventions can significantly reduce loneliness, particularly among those studies involving communication, gaming, or virtual pet companions (13). A PVA can become a companion that one can actually communicate and/or play games with, and be entertained by (43). Participants were able to successfully use the device without major problems, completing an average of 18 daily interactions with the device in the first 4 weeks when a minimum of five were required, and an average of 10 daily interactions in the second 4 weeks when no minimum interactions were required.

Our findings also demonstrate that anthropomorphism of PVAs through relational greetings mitigated loneliness and baseline loneliness predicted relational greetings with the PVA. As noted earlier, anthropomorphism is often conceptualized as the attribution of human traits to non-human entities (20) and anthropomorphic interactions are typically driven by a user's desire to make social connections and form relationships with non-human entities (20, 28). The limited prior research that has been done about anthropomorphism and PVA use has supported how socioemotional states such as loneliness can drive anthropomorphism, and how polite terms and behaviors such as “please,” “thank you,” and “good afternoon” reflect personification of the device (16). This may be particularly useful information to positively impact loneliness in the cohort of adults aged 75+, which is representative of the sample used in our study.

While the coded usage data clearly indicate that participants were engaging with the device as an anthropomorphic agent in many different ways (i.e., reactions, polite language, comments/questions, and greetings), participants who were more lonely were more likely to seek out interactions with the PVA (i.e., not required to use the device) and initiate personal greetings (e.g., “Good morning,” or “how are you today?”). These findings may be rooted in time of day and social activity; greetings/goodnights may be a distinct way by which more lonely individuals seek connection at times of day when the home is most likely to be empty. Greetings and goodbyes also represent interaction rituals, identified by Goffman as integral elements of social interaction, demonstrating regard and respect for those interacting (52, 53). When interacting with the PVA device, the user's willingness to follow social norms of politeness and interaction rituals indicates her/his respect for subtle social nuances when entering a relationship with the device. Relational closeness related behaviors, such as “Alexa, I am leaving. You're in charge of the cat now,” are clear signs of a user's desire to personally connect with the device. All of such behaviors are indications of a user's desire to connect with the PVA on a personal level.

### Limitations and Future Studies

Taking a step further from prior studies that examined how older adults interact with a PVA (33, 35), the current project investigated the impacts of such PVA interactions on health and quality of life outcomes by exploring how loneliness in older adults could be influenced by anthropomorphic interactions with a PVA. To our knowledge this was the first study to explore how loneliness in adults 75+ could be influenced by anthropomorphic interactions with a PVA. Focusing on PVA use among the “older old” of participants 75+ makes our data a valuable addition to prior studies that included “younger old” participants, aged 65+ (33, 35) and should be of interest to researchers and practitioners interested in gerotechnology during this advanced stage of aging. Due to practical reasons of recruiting in a small pool of advanced ages of older adults, i.e., 75 or older, we adopted a single-group quasi-experimental design, which is within the norm of technology-based health intervention studies (54). Since research recruitment was halted due to the COVID-19 pandemic, future studies should include a larger diverse sample, and a comparison group with PVA use over a longer time period. Use of the PVA could also include different types of interactions, incorporating interaction types that are evidence-based from prior loneliness interventions, and those that are personalized to the user. Like prior research, the participants in our study were largely female older adults (55). Future investigations should examine whether men and women interact with PVA devices in different ways, and whether this has a variable impact on loneliness. Although our sample included men and women, the sample size was too small to allow for meaningful consideration of potential differences.

This study used living alone as a proxy for potential loneliness, and did not screen out participants based on their levels of loneliness. It is possible that some individuals in this study were simply not that lonely; they have many opportunities for social interaction living in a community residence. Despite this, results still show a consistent effect for the PVA on loneliness perceptions. Future research can address this gap by adding inclusion/exclusion criteria for those who have increased loneliness. Finally, the unique study design, incorporating a combination of device usage data, repeated surveys over time, and real-world location in the homes of individuals rather than in a lab, also demonstrates potential for understanding ICT use and influence moving forward.

## Conclusion

Our study breaks new ground by showing the direct impact of PVA anthropomorphization on loneliness among this understudied, older 75+ population. Results indicate that as the aging adult anthropomorphizes PVAs, her/his loneliness subsides, illustrating a novel effect of baseline loneliness as an impetus for aging adults to anthropomorphize PVAs, perhaps as a mechanism to chip away isolation in her/his life. Our data demonstrate how engaging with an affordable, out-of-the-box technological innovation like the Amazon Echo can help reduce loneliness in older adults. It further suggests that the “older old” of 75+ year olds can have positive attitudes toward and demonstrate interest in using technological innovations to deliver interventions. In fact, many participants continued to use the device through the COVID-19 pandemic, demonstrating its potential as a longer-term loneliness intervention. While there is no “one size fits all” approach to addressing loneliness, and this type of intervention likely isn't right for everyone, the opportunity for participants to exercise control over and individualize the experience, drawing on thousands of commands and capabilities the Alexa AI provides, may enable a type of tailoring to the user that other technological innovations aren't able to provide.

## Data Availability Statement

The datasets presented in this article are not readily available because we do not have IRB approval to share the data. Requests to access the datasets should be directed to valeriejones@unl.edu.

## Ethics Statement

The studies involving human participants were reviewed and approved by University of Nebraska Institutional Review Board. The patients/participants provided their written informed consent to participate in this study.

## Author Contributions

VJ and MH developed the grant and study, collected the data, and crafted the manuscript, with CY analyzing and interpreting data and results and contributing to the manuscript. MS and JB provided valuable insight, additions, and assistance on the manuscript. RMB provided great contributions as well, particularly in coding and interpreting the data. All authors contributed to the article and approved the submitted version.

## Funding

Funding for this study was provided by a University of Nebraska-Lincoln Layman Award.

## Conflict of Interest

The authors declare that the research was conducted in the absence of any commercial or financial relationships that could be construed as a potential conflict of interest.

## Publisher's Note

All claims expressed in this article are solely those of the authors and do not necessarily represent those of their affiliated organizations, or those of the publisher, the editors and the reviewers. Any product that may be evaluated in this article, or claim that may be made by its manufacturer, is not guaranteed or endorsed by the publisher.
