# Supplementary material for: Reducing Loneliness Among Aging Adults: The Roles of Personal Voice Assistants and Anthropomorphic Interactions
Source: Front Public Health. 2021 Dec 10;9:750736. doi: 10.3389/fpubh.2021.750736 (PMC8702424; doi:10.3389/fpubh.2021.750736)
Supplement: Supplementary file 1 [file Data_Sheet_1.PDF]

Using Your Alexa Assistant

This "menu" of commands can help you get started

This Echo Dot is a device made by Amazon. It is designed for you to speak to it, ask it questions and give commands. An artificial intelligence (AI) assistant inside of it, called "Alexa," powers the responses and takes actions at your command.

This list was originally developed for a research study at the University of Nebraska by Dr. Valerie Jones, focused on helping aging adults use Alexa, and is being made available for anyone to use.

This list includes a variety of commands to use with Alexa, the personal assistant built into your Echo Dot device. Feel free to try your own commands, too.

To start, just say "Alexa," and then the ring of the Echo Dot device will light up in blue and Alexa will start listening for your request. **You must say "Alexa" to make it work.**

At any time, tell Alexa to stop what it's doing by saying "Alexa, stop." You may need to say "Alexa, stop" before it can start doing something else.

Alexa will only record what you say to Alexa, and not other conversations you have in your home. Since skills change and are updated regularly on Alexa, it's possible that some may not be available. That's why there are many commands to choose from.

Basic/ Information Retrieval

Getting Everyday Information

- "Alexa, what's today's date?"
- "Alexa, what time is it?"
- "Alexa, what's the weather like?"
- "Alexa, will it rain today?"
- "Alexa, what will the weather be like tomorrow?"
- "Alexa, what's the traffic like?"
- "Alexa, what time is it in [city or country]?"
- "Alexa, what's yesterday's [team name] score?"
- "Alexa, when is the next [team name] game?"
- "Alexa, how do you spell [word]?"
- Alexa, what's another word for [word]?
- "Alexa, who was the 7th president of the United States?"
- "Alexa, what's the stock report?"
- "Alexa, what's the stock price of [Berkshire Hathaway]?"
- "Alexa, open Opening Bell."

News, Information, and Questions and Answers

- "Alexa, what's in the news?"
- "Alexa, play my flash briefing."
- "Alexa, how's traffic to XYZ?"
- "Alexa, what are synonyms for "helpful"?"
- "Alexa, how do you say "thank you" in Italian?"

Entertainment

- "Alexa, play NPR or play NET radio"
- "Alexa, play [radio station]."
- "Alexa, play sports radio."
- "Alexa, what movies are playing?"
- "Alexa, tell me about the movie [whatever it's called]"
- "Alexa, what is the IMDB rating for [show/movie]?"
- "Alexa, who stars in [show/movie]?"
- "Alexa, what's playing on TV tonight?"
- "Alexa, sing me a song."
- "Alexa, tell me a joke."
- "Alexa, tell me a riddle."
- "Alexa, tell me a poem."
- "Alexa, read my Kindle book." (If you purchase a book on a Kindle, Alexa will read it aloud to you.)
- "Alexa, play an Audible book."
    - "Alexa will play a free audiobook, such as The Hobbit, as a trial from Audible.com"
    - "Alexa, resume my Audible book."
- "Alexa, play [artist name] or [song name]."
- "Alexa, louder."
- "Alexa, softer."
- "Alexa, stop."
- "Alexa, launch This Day in History."
- "Alexa, open Curiosity."
- "Alexa, open BrainFacts."
- "Alexa, open Today's Word of the Day."

Cooking

- "Alexa, how do you make [recipe]?"
- "Alexa, how many tablespoons are in a quarter cup?"
- "Alexa, what's a substitute for [heavy cream]?"
- "Alexa, what wine pairs well with [name of food]?"

Health

- "Alexa, help me sleep."
- "Alexa, open guided meditation."
- "Alexa, open HeadSpace."
- "Alexa, start SpaMusic."
- "Alexa, start Daybreak Yoga."
- "Alexa, open 6-Minute Full Body Stretch."

Encouragement/ Motivation/ Religion

- "Alexa, open Daily Morning Affirmations"
- "Alexa, what is the Verse of the Day?"
- "Alexa, play our Daily Bread"
- "Alexa, give me a quote from Daily Quotes."

The University of Nebraska does not discriminate based upon any protected status. Please see go.unl.edu/nondiscrimination.
©2021

Interaction

Making and Taking Calls

- This only works if you have the Alexa app on your smartphone.
- "Alexa, call Jerry" (or anyone on your smartphone contacts list)
- "Alexa, answer the call/ hang up"

Conversation

- "Alexa, let's chat"
  This calls up one of many different "social bots," or conversation programs, that have been built by teams of computer scientists around the world, and enables Alexa to have a conversation with you.

Games

(Many of these can be played by yourself or with a friend)

- "Alexa, open Trivia Hero."
  This is a trivia game that can be played yourself or with others in which you answer as many trivia questions as you can in a certain amount of time.
- "Alexa, play True or False."
  You can play yourself or with someone else. Alexa asks you a random question and you try to answer it correctly. If playing yourself, Alexa sometimes tells you how you did compared with other people who played.
- "Alexa, play Would You Rather."
  Alexa asks you if you would rather do one of two different things, and may compare your responses with others it has gotten from other players.
- "Alexa, open Guess My Name." You can choose lightning round, head-to-head, or multi-player. You have two minutes to get as many questions right as you can, guessing the name of the person Alexa describes.
- "Alexa, play Spelling Bee." (Can be played alone or with friends)
  Alexa randomly selects words for you to try to spell.

Games continued

- "Alexa, open Jeopardy!"
  This is modeled after the popular game show
- "Alexa, open Question of the Day."
  Alexa asks a random question; you try to answer it correctly to earn points, and it tells you how you compare with others.
- "Alexa, open 20 Questions."
  Respond to questions it asks as it tries to identify what you're thinking of.
- "Alexa, open Math Puzzles."
  You try to figure out the next number in the sequence.
- "Alexa, open the Magic Door."
  This is an interactive, choose-your-own-adventure type of story.
- "Alexa, play Song Quiz."
  You'll give a name, location, and decade for the songs, and you try to name the song and artist. Alexa will find an opponent for you to play.
- "Alexa, open Wait Wait Quiz."
  This is modeled after the "Wait Wait, Don't Tell Me" quiz show on NPR, and asks you questions about current events.
- "Alexa, open Game of Lists." (Good to play with a group)
  Alexa challenges you to write down as many things as you can in 30 seconds for a category it randomly selects, and to try to get more things on your list than someone else.

The University of Nebraska does not discriminate based upon any protected status. Please see go.unl.edu/nondiscrimination.
©2021

Personalization/Customization

Create and Add to Different Types of Lists

- "Alexa, create a [use any example below] list."
    - To do list
    - Vacation packing list
    - Holiday gift list
    - Shopping list
    - Grocery list
- "Alexa, add hair cut to my to-do list"
- "Alexa, what's on my to-do list?"
- "Alexa, clear my to-do list."
- "Alexa, add an item to my holiday gift list"
- "Alexa, what's on my holiday gift list?"
- "Alexa, remove item from my holiday gift list"
- "Alexa, add item to my vacation packing list"
- "Alexa, add butter to my grocery list"
- "Alexa, add paper towels to my shopping list."
- "Alexa, what's on my shopping list?" or "grocery list"

Timer, Alarm, Reminder Commands

- "Alexa, set alarm for [time]"
- "Alexa, wake me up every day at [time]"
- "Alexa, wake me up at [time] to [music or radio station]."
- "Alexa, snooze" [For nine minutes]
- "Alexa, set timer for [X minutes]."
- "Alexa, set a 10 minute [pizza] timer." (If using multiple timers, Alexa will tell you your "pizza is ready".)
- "Alexa, how long is left on the timer?"
- "Alexa, stop the timer"
- "Alexa, set a reminder every day at 7 pm to [water my plants] [call my neighbor]."
- "Alexa, set a reminder for my XYZ appointment on XYZ date"
- "Alexa, remind me next Tuesday that it's Mike's birthday"
- "Alexa, remind me to start the laundry at 2 pm."
- "Alexa, remind me to take my [medication type] every day at 9am."
    - If you have multiple medications that need to be taken at different times, then you can set reminders for each particular medication.
- Need a reminder of your reminders? Just say, "Alexa, what are my reminders?" Your Echo will announce them out loud.

Checking Calendars and Reminders

- "Alexa, what's on my schedule?"
- "Alexa, add an event on my calendar"
- "Alexa, add birthday lunch to my calendar on Thursday at noon"
- "Alexa, add committee meeting to my calendar on November third at 2:30"
- "Alexa, what am I doing tomorrow?"

Skills

Skills are basically different programs Alexa can run and use.

Skills

"• Alexa, open Ted Talks"

- "Alexa, open Radio Mystery Theater"
- "Alexa, ask Ted Talks to find talks about nature"
- "Alexa, open Entertainment Weekly."

Sports

- "Alexa, enable Golf Channel"

Sleep/Relaxation

- "Alexa, play Ambient Sounds"
    - "Alexa, turn this off in 10 minutes"
- To enable: "Alexa, play Ocean Sounds"
- To enable: "Alexa, enable Mindfulness" or visit the Alexa Skills Store
- Top command: "Alexa, ask Mindfulness for a peaceful meditation"
- "Alexa, help me get started with skills." Alexa will suggest skills you might like to try.

The University of Nebraska does not discriminate based upon any protected status. Please see go.unl.edu/nondiscrimination.
©2021
